# Increased risk of psychosis to ivermectin treatment

**DOI:** 10.1192/j.eurpsy.2022.2067

**Published:** 2022-09-01

**Authors:** A. Romero Calderon, M. Marín Valdovino, A.M. González Meléndez, J. Leiva Soto, A. Garcia Baltazar

**Affiliations:** 1 HOSPITAL SAN JUAN DE DIOS, EnseÑanza, ZAPOPAN, Mexico; 2 Hospital San Juan De Dios, Psiquiatria, Zapopan, Mexico

**Keywords:** Ivermectin, Psychosis

## Abstract

**Introduction:**

Adolescence: a state of mental fragility, where psychiatric disorders may debut, where have been reported several cases of toxidermy, encephalopathy and neuropsychiatric disorder related to ivermectin treatment (excluding organicity, substance abuse or medication) (1). It has effect on the dopaminergic system with an alteration on glycoprotein P leading to high levels of ivermectin causing neurotoxicity (2). In this poster, we discuss the case of a 14 y/o´s psychosis to ivermectin

**Objectives:**

Presentation of a clinical report

**Methods:**

14 years old woman. Background: father with schizophrenia. Comorbility: None. Initially, presented to ER with 2 week of treatment to ivermectin 6mg for pediculosis; presenting first psychotic episode. She presented first clinical outbreak of psychosis characterized by mystical-religious, erotomaniac, harm and reference delusions, auditory and visual hallucinations adding isolation, abulia, apathy, dialogued soliloquies, and spontaneous crying. No prior psychiatric treatment.

**Results:**

In hospitalization: elevated indirect and direct bilirubins and hiponatremia; neuroimaging studies are reported normal. Haloperidol 7.5 mg/day is indicated and parkinsonism is presented. Treatment is changed to olanzapine 15mg/day with notorious improvement. Diagnostic impression: Acute polymorphic psychotic disorder with symptoms of schizophrenia

**Conclusions:**

Possible causes were analyzed, finding a relationship with ivermectin treatment. This case makes evident the importance of conducting in-depht evaluations and finding risks factors for psychosis.

**Disclosure:**

No significant relationships.

